# Distinct Expression of Immunoglobulin-Binding Proteins in Shiga Toxin-Producing *Escherichia coli* Implicates High Protein Stability and a Characteristic Phenotype

**DOI:** 10.3390/toxins9050153

**Published:** 2017-04-29

**Authors:** Dennis Rubin, Wenlan Zhang, Helge Karch, Thorsten Kuczius

**Affiliations:** Institute for Hygiene, Westfälische Wilhelms-University and University Hospital Münster, Robert Koch-Straße 41, 48149 Münster, Germany; dennis.rubin@ukmuenster.de (D.R.); wenlan.zhang@ukmuenster.de (W.Z.); hkarch@uni-muenster.de (H.K.)

**Keywords:** regulation, expression, immunoglobulin-binding protein G, Shiga toxin-producing *Escherichia coli*

## Abstract

Several immunoglobulin-binding proteins of *Escherichia coli* (Eib) have been isolated from both non-pathogenic and pathogenic *E. coli* strains. Shiga toxin (Stx)-producing *E. coli* (STEC) contain *eib*G either as a single gene or in combination with *eib*C, while other *E. coli* strains harbour single or multiple *eib* genes. The Eib proteins bind human immunoglobulins in a non-immune manner and contribute to bacterial chain-like adherence to human epithelial cells. In this study, the EibG expression in several STEC strains was analysed under different environmental conditions. STEC produced high levels of EibG in complex media and lower levels in low-grade and minimal media under static growth conditions. This characteristic was independent on the Eib subtypes. Microscopically, EibG-expressing STEC exhibited chain formation and aggregation in all employed media, while aggregates were only visible after growth in complex medium. Once expressed, EibG proteins demonstrate high stability during prolonged incubation. Our findings indicate that the regulation of the expression of Eib proteins is highly complex, although the protein levels vary among STEC strains. However, positive upregulation conditions generally result in distinct phenotypes of the isolates.

## 1. Introduction

Immunoglobulins are involved in mammalian immune responses. Several bacteria capable of causing severe infectious diseases have developed defence strategies allowing them to survive in the host by synthesizing surface proteins that interact with immunoglobulins in a non-immune manner [1]. Such immunoglobulin-binding proteins were identified within different bacterial species and in commensal *Escherichia coli* (Eib) may play a role in virulence and are able to impart resistance to human serum complement [2,3]. Some strains associated with the *E. coli* standard collection of reference (ECOR) [4] and isolates from cervids carry one gene including *eib*F or *eib*H, respectively [5,6], but other ECOR strains contain up to several genes as *eib*A, *eib*C, *eib*D, and *eib*E [3]. Eib proteins were also detected in Shiga toxin (Stx)-producing *E. coli* (STEC) [7] with *eib*G occurring as a single gene or in combination with *eib*C [8]. Three different EibG subtypes (EibG-α, EibG-β, and EibG-γ) have been identified among human STEC isolates [7].

STEC are highly diverse with respect to their phylogeny, combination of virulence factors, reservoirs, disease association, and epidemiology [9]. The main STEC virulence characteristics are the toxins Stx1 and Stx2 [10]. Human infection by STEC is manifested as uncomplicated diarrhoea, severe haemorrhagic colitis, or the haemolytic uremic syndrome (HUS). Current models suggest that during HUS, Stx preferentially binds to microvascular endothelial cells of the renal glomeruli and the brain [11]. In addition, these toxins are also capable of activating cell stress signalling pathways, which can lead to apoptosis, autophagy, and activation of the innate immune response [12]. Moreover, the recently observed direct toxicity of Stx towards developing human erythrocytes during the course of erythropoiesis might contribute to the pathogenesis of HUS, in particular to the HUS-associated anaemia [13]. Additional STEC virulence factors include the adhesin intimin, EHEC haemolysin, cytolethal distending toxin, a catalase-peroxidase and an extracellular serine protease (EspP) [14,15,16,17,18,19,20,21], which occur in various combinations. However, STEC strains producing EibG lack intimin [7]. EibG was shown to contribute to the adherence of intimin-negative STEC to the host intestinal epithelial cells and to confer a typical chain-like adherence pattern (CLAP) [7]. Bacteria expressing EibG exhibit phenotypes, such as cell aggregation and chain-like adherence patterns to bacterial cells [8] and host epithelial cells [7,22], as well as biofilm formation [8].

In SDS-polyacrylamide gels, EibG formed heat-stable multimeric proteins with dominant signals of a molecular mass >250 kDa and a band at approximately 120 kDa with lower intensity [8], whereas the predicted mass of the largest Eib monomer is approximately 54 kDa [3]. All these multimeric isoforms are able to interact non-specifically with the immunoglobulins IgA and/or IgG and with the Fc fragment of human IgG [2]. In contrast to very faint signals after growth under shaking conditions, high protein expression was detected under static growth of EibG-positive STEC isolates [8]. This indicates a positive regulation of expression, i.e., that high levels may be produced when the proteins are required. However, the levels of EibG proteins varied among the isolates from strong to distinct signals, but faint intensities under growth conditions provide high expression rates. One reason for the differential gene product levels might be the stability of the proteins. In particular, increased EibG levels argue in favour of protein stability over a prolonged incubation time, whereas instable proteins would decrease in signal intensity. Furthermore, varied cultivation conditions might have an impact on EibG expression levels. In this study, we followed the expression and stability of EibG proteins from STEC and analysed growth conditions, which facilitate the up- and downregulation of EibG proteins. We compared the EibG regulation with those of other immunoglobulin-binding proteins in *E. coli* by using the strains ECOR2 harbouring *eib*F [5] and ECOR9 carrying four different *eib* genes (*eib*A, *eib*C, *eib*D and *eib*E) [3].

## 2. Results

### 2.1. EibG Is Highly Upregulated Under Static Incubation, yet the Protein Is Differentially Expressed under Shaking and Non-Shaking Conditions among Isolates

**Figure 1:**
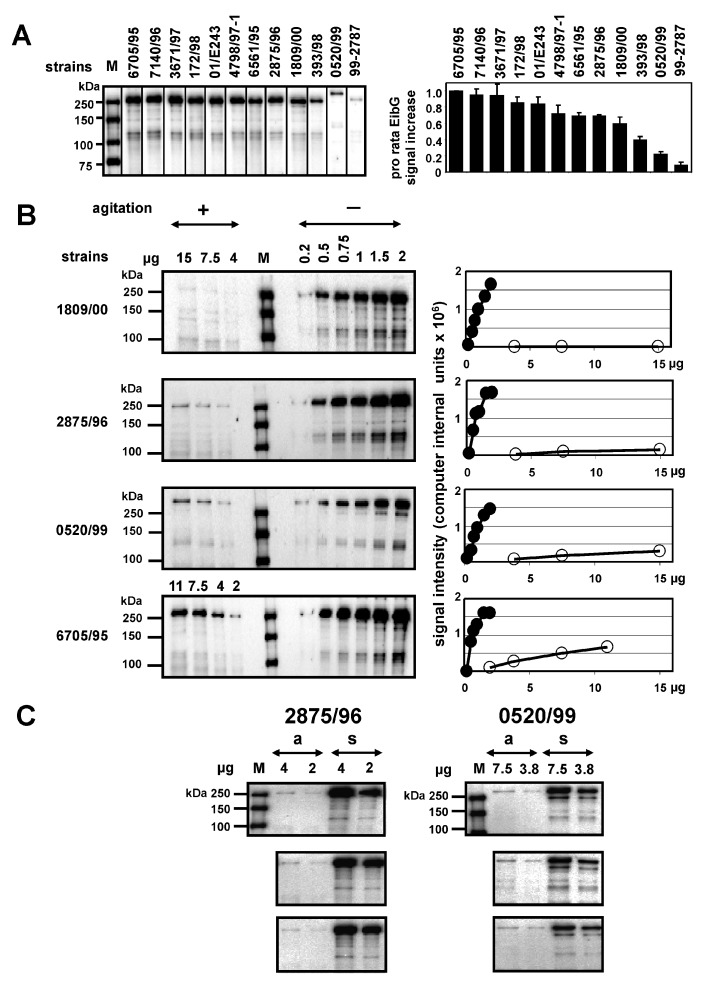
Differential EibG protein levels detected in various STEC by immunoblotting. Proteins derived from EibG-positive STEC strains were visualised on immunoblots using HRP-conjugated IgG Fc fragments and EibG signal intensities were quantified by densitometry. (**A**) After cultivation without agitation, proteins from the STEC strains (5 µg each) were separated, immunoblotted (left graphic) and EibG specific signal intensities were calculated (right graphic). To control for variations in the methodology, at least three independent immunoblots were included into each calculation. For comparison, EibG signal intensities of STEC representing the highest signals on an immunoblot were defined as 1.0. Variations of repeated SDS-PAGE runs were expressed as standard deviations (±SD of the means). (**B**) EibG positive STEC were cultivated with (+) and without (−) agitation. Proteins were finally separated in dilutions as indicated by SDS-PAGE, immunoblotted, and specific proteins were visualised immunologically (left blot). EibG signals were quantified using the imager technique (right graphic). Black and white circles represent EibG signal intensities after static and shaking conditions, respectively. (**C**) To demonstrate reproducibility of the high and low expression of various EibG subtypes, colonies from the wild-type strains carrying the α-type and the γ-type were cultivated in three independent probes with agitation at 180 rpm (a) and under static growth conditions (s) at 37 °C. For precise identification different protein amounts were loaded onto gels as 4 µg and 2 µg for strain 2875/96 (α-type) and 7.5 µg and 3.8 µg for strain 0520/99 (γ-type), separated electrophoretically and immunoblotted. Sizes of the marker proteins are indicated (M).

STEC harbouring *eibG* produced high levels of EibG under static growth conditions in the complex Luria-Bertani (LB) medium. This is in contrast to agitated cultivation, which resulted in very faint bands on immunoblots or protein levels below the detection limit (Figure 1). In order to analyse differential EibG expression levels among isolates, STEC were cultivated under static conditions. Identical concentrations of proteins were loaded onto SDS gels prior to immunoblotting using the human IgG Fc fragment, and signals were measured (Figure 1A). EibG proteins consist of a multimeric complex. The average identity sequence coverage for the EibG α-subtype of strain 2875/96 was 39% (data not shown). STEC showed EibG proteins mostly as multimers with a dominant EibG band at a molecular mass >250 kDa. Signals of isoforms with lower masses were also detectable: predominantly, a distinct band was observed at approximately 120 kDa. Proteins with lower masses produced very faint bands or were below the level of detection primarily when the strains generated low levels of EibG proteins and may, therefore, be disregarded. Strain 6705/95 demonstrated the highest EibG signal intensities. Strains 7140/96 and 3671/97 demonstrated only slightly reduced levels (Figure 1A). In contrast, low and weak EibG signals were detected in STEC 393/98 and 99-02787 as well as in strain 0520/99 (Figure 1A), the latter containing, as the only one among the strains tested, *eibG* of γ-subtype (Table 1).

These differential EibG expression levels among the strains detected under identical static growth conditions led to the assumption that some STEC would produce more EibG proteins than others. Therefore, we examined EibG signals under shaking and static conditions and found that high signals detected after static growth did not correlate with highly-expressed signals under shaking conditions. For precise identification two different protein amounts were loaded onto gels for strain 2875/96 (α-type) and for strain 0520/99 (γ-type), respectively, for separation and immunoblotting. The bacteria demonstrated a reproducible strain-specific pattern with very similar signal intensities. Despite the fact that increased protein concentrations were loaded onto gels, EibG signal intensities displayed by shaken cultures were faint and near the detection limit in strains 1809/00 and 2875/96, both of which express the α-subtype, and 0520/99, which produces the γ-subtype of EibG (Figure 1B). On the other hand, considerably higher signals under shaking conditions were identified in strain 6705/95 (Figure 1B). These various EibG levels expressed by STEC were specific and reproducible for particular strains as shown for 2875/96 and 0520/99 (Figure 1C).

### 2.2. Impact of Medium Composition on EibG Expression in STEC and Other Eib Proteins in E. coli

**Figure 2:**
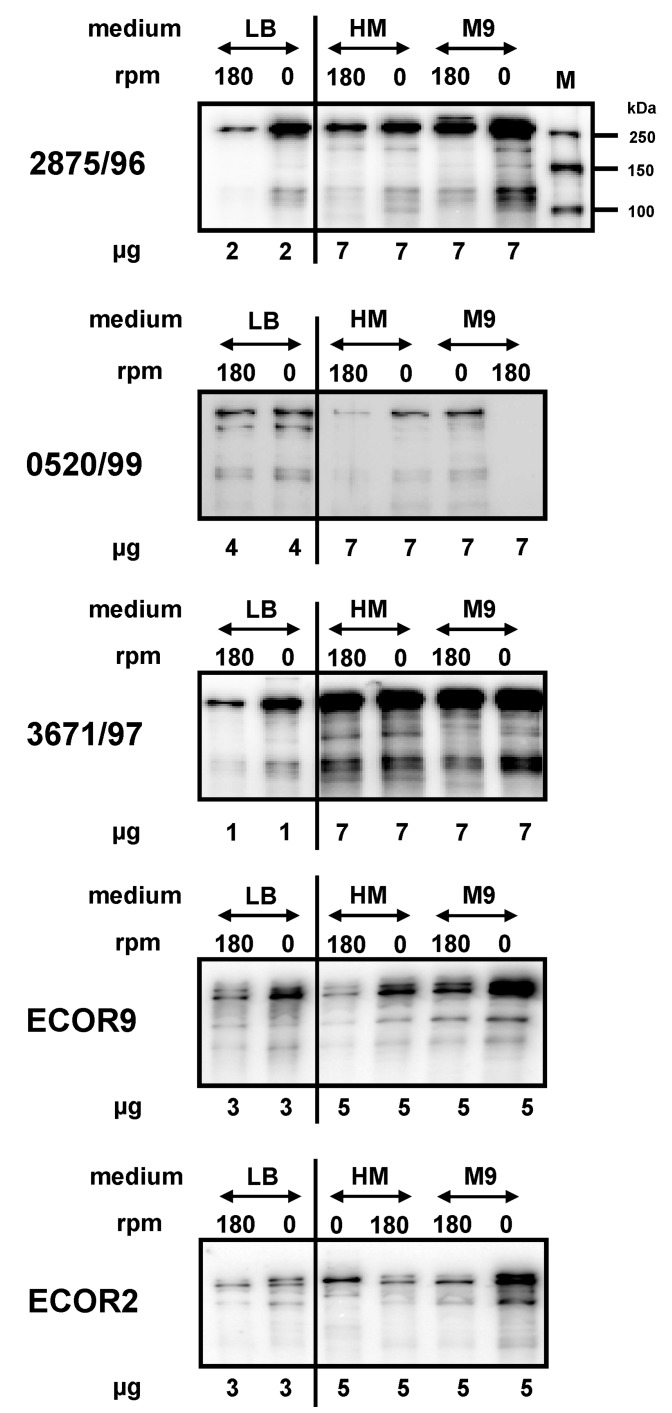
The composition of the growth medium has an impact on EibG protein expression in STEC and on other Eib proteins in *E. coli.* EibG-positive STEC, as well as ECOR2 and ECOR9, were cultivated with agitation at 180 rpm and under static growth conditions (0 rpm) at 37 °C in the following media: LB broth as a complex medium, yeast extract medium (HM) and minimal medium (M9). For the distinctive differentiation of EibG expression levels, various protein amounts (in the graph interrupted by a line) of 2 µg and 7 µg for strain 2875/96 (α-type), 4 µg and 7 µg for strain 0520/99 (γ-type), and 1 µg and 7 µg for strain 3671/97 (harbouring genes *eib*C and *eib*G) were separated and immunoblotted, as indicated earlier. For ECOR2 and ECOR9 strains, 3 µg and 5 µg of proteins were loaded, respectively. Sizes of the marker proteins are indicated (M).

The considerably varying interstrain EibG levels may be a result of additional regulatory factors, such as temperature and oxygen, which generally have the potential to downregulate [8], as well as media composition. To gain insight into the latter issue, we analysed the impact of the composition of the growth media on EibG expression in STEC. In the complex medium such as LB, the highest EibG levels were detected in static cultures and low levels in shaken cultures of STEC 2875/96, 0520/99, and 3671/97, the latter of which also harbours the *eib*C gene. Cultivation in the minimal medium (M9) and in the yeast extract medium (HM) resulted in a considerable decrease in the level of EibG. To achieve sufficient signal intensities, increased protein amounts had been loaded onto immunoblots (Figure 2). Despite low expression, the impact of agitation vs. static incubation on the up- and downregulation of EibG was detectable; signals became more visible when cells were grown in the absence of shaking. In all tested media, static growth resulted in increased EibG synthesis compared to growth with agitation (Figure 2). All strains demonstrated highest Eib expression levels after static growth in the complex medium followed by incubation in the minimal medium.

**Figure 3:**
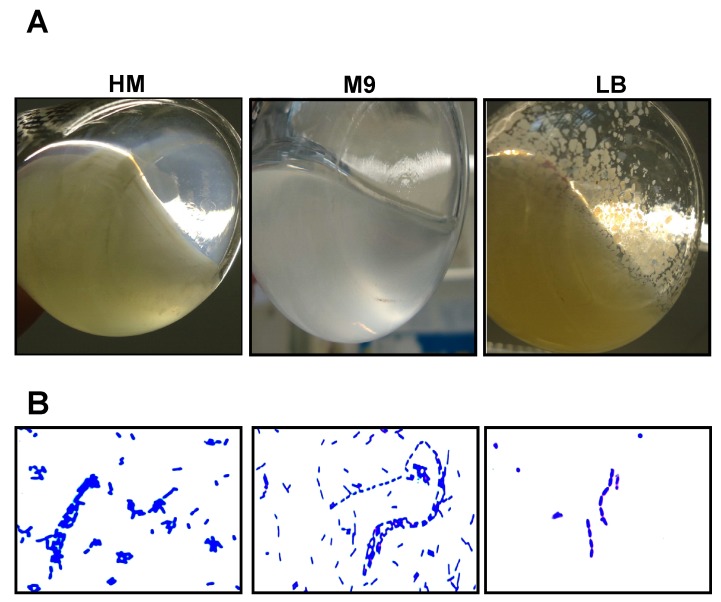
Diverse phenotypes are displayed by EibG-producing strain 2875/96 in different nutrient media. Strain 2875/96 was incubated without shaking at 37 °C for 20 h. (**A**) Bacteria aggregated and formed deposits in the complex media (LB). In reduced media, such as the yeast extract medium (HM) and the minimal medium (M9), cells grew homogeneously. (**B**) Microscopically, however, cells exhibited aggregates and clumps in all media. Figures show images after crystal violet staining.

Bacteria expressing EibG exhibit distinct phenotypes [7,8,22]. STEC 2875/96 bacteria formed aggregates, appeared as clumps (Figure 3A) and formed chain-like patterns when grown in the complex media without shaking (Figure 3B). Interestingly, these aggregates were not identifiable after cultivation in the yeast extract medium and the minimal medium at the macroscopic level. Microscopically, however, aggregated cells were detected as chains and clumps (Figure 3B).

The protein expression data presented above were obtained with STEC strains harbouring the *eib*G gene. However, other immunoglobulin-binding proteins were also identified in other *E. coli* strains. Specifically, in the ECOR collection [4], ECOR9 harbours four genes (*eib*A, *eib*C, *eib*D, and *eib*E) [3], whereas ECOR2 carried only the *eib*F gene [5]. These proteins demonstrated characteristic multimerization similar to EibG in STEC. All products were identified as multimeric complex proteins with large molecular masses >120 to >180 kDa in denaturing SDS-PAGE and immunoblots using the Fc fragment of human IgG as detection antibody [2]. We analysed the expression of several Eib proteins, such as EibA, EibC, EibD, and EibE in ECOR9 and EibF in ECOR2. We compared the factors influencing up- and downregulation of these Eib proteins in various media with that of EibG in STEC. Overall, we observed very similar regulation of expression. The non-EibG Eib proteins in ECOR strains were produced in high concentrations under static growth. The highest levels were detected in the complex medium, and decreased levels in the minimal and yeast extract media, respectively. The impact of the medium composition and culture conditions on the expression of non-EibG Eib proteins was, thus, similar to that on EibG synthesis (data not shown).

### 2.3. Correlation between Bacterial Growth and EibG Expression in STEC under Different Growth Conditions

**Figure 4:**
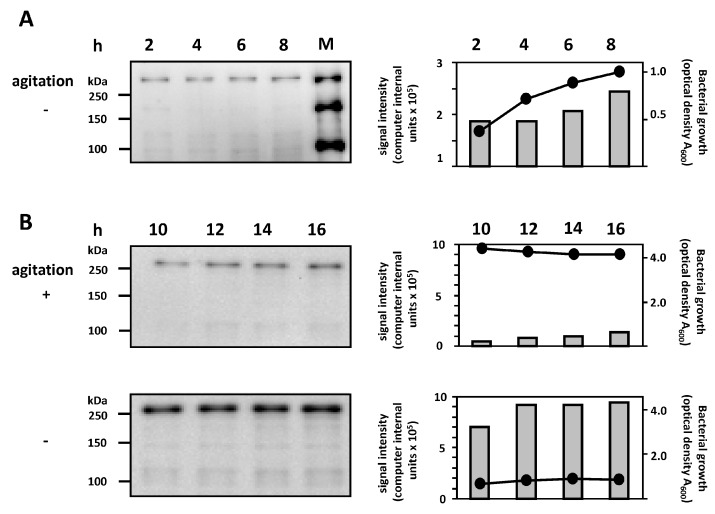
EibG is highly expressed in the stationary phase under static growth conditions. Strain 2875/96 was incubated under static growth conditions (− agitation) and with shaking (+ agitation). Bacterial growth and protein content were determined at different time intervals as indicated. Protein aliquots of 3 µg (**A**) and 2 µg (**B**) per lane were immunoblotted and EibG signals were visualized using HRP-conjugated human IgG Fc fragment. Molecular masses of the standard proteins are indicated. Growth of bacteria was measured photometrically and followed by values of the optical density (closed circles and black lines) of these exemplary setups for short (**A**) and long incubation (**B**). Grey bars represent the signal intensities of EibG given as computer internal units. The results represent one out of three independent curves.

We further compared the EibG expression in strain 2875/96 with the bacterial growth under different growth conditions. Under static incubation in the complex LB medium, a correlation between the growth of STEC strain 2875/96 and gradually increasing EibG expression was observed within the first 8 h; at the stationary phase, both the bacterial growth and EibG level reached a plateau (Figure 4A,B). Incubation under shaking conditions resulted in high bacterial density in the stationary phase, but EibG expression was low (Figure 4B). These experiments demonstrated that STEC expressed high EibG levels in the stationary phase under static incubation.

### 2.4. Regulation and Stability of EibG Proteins in STEC

**Figure 5:**
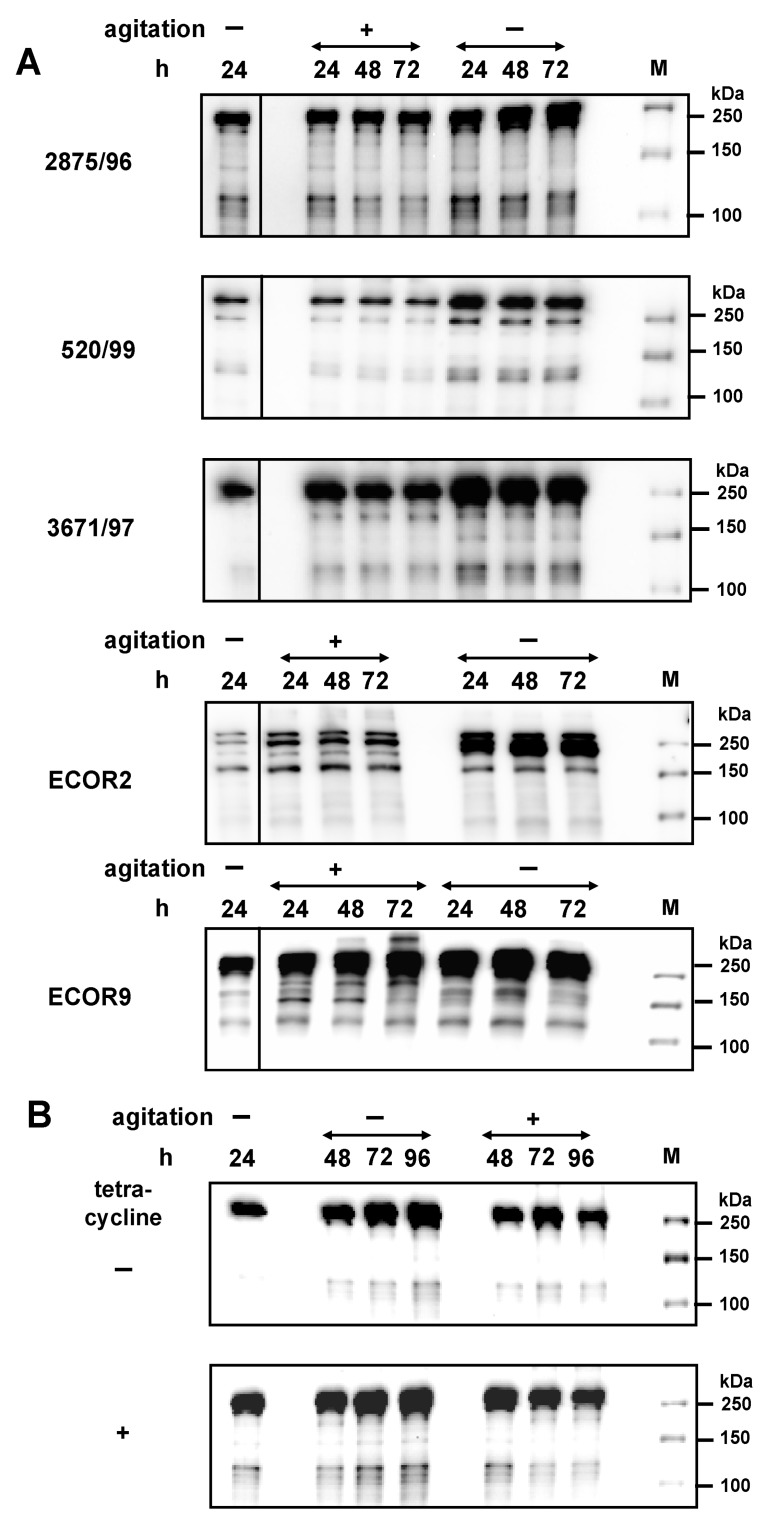
Once expressed, EibG proteins demonstrate high stability during prolonged incubation. For the adequate synthesis of EibG, strains 2875/96, 0520/99, and 3671/97 were incubated statically in LB broth without agitation (−) for 24 h. (**A**) These cultures were re-inoculated into a fresh medium at a 1:100 dilution followed by incubation with (+) and without (−) shaking. Aliquots were harvested after indicated time periods. Proteins (4 µg per lane) were immunoblotted and EibG were detected using human IgG Fc fragment antibody. (**B**) After static growth for 24 h and expression of EibG, the culture of STEC strain 2875/96 was aliquoted and incubation was extended with (+) and without (−) shaking. To inhibit protein and EibG synthesis, one aliquot was laced with tetracycline (20 µg/mL) (+). Cells were harvested at the indicated periods of time and proteins from the aliquots were immunoblotted. Molecular masses of standard proteins are indicated.

Increased Eib protein expression under stress conditions, such as high cell densities, high amounts of metabolic waste products, or pH changes may increase the stability of these proteins. Therefore, we analysed the stability of the synthesized EibG proteins over a prolonged incubation time. For high expression of Eib proteins, STEC strains 2875/96, 0520/99, and 3671/97, as well as ECOR2 and ECOR9 were pre-incubated statically up to the late stationary phase (Figure 5A). These pre-cultures were then sub-cultivated into fresh complex media followed by incubation for 24 to 72 h with and without shaking. Eib protein signals as high as in the pre-culture were measured when STEC isolates and ECOR strains were incubated for extended time (Figure 5A). This result indicates that Eib proteins were not degraded by internal enzymatic activities or other regulatory mechanisms during incubation that downregulate protein levels. On the other hand, STEC and ECOR, which grew under static conditions, gave rise to increased Eib expression with signals increase generally within the first 24 h. After that, Eib remained at this high signal level during prolonged cultivation, up to 72 h (Figure 5A).

Furthermore, the stability of the synthetized Eib was demonstrated by extended incubation for up to 96 h. High Eib levels were measured after static overnight incubation followed by extended incubation up to 96 h with and without shaking (Figure 5B). The EibG signals remained high and stable, independent of cultivation conditions. To confirm that the Eib signals did not result from de novo Eib production, the antibiotic tetracycline, which binds to ribosomes and inhibits protein synthesis, was added. The Eib signals in the tetracycline-treated cultures were as high as those in untreated culture even after an extended incubation time of 96 h (Figure 5B). Hence, the high Eib signals observed up to 96 h of incubation did not result from de novo Eib synthesis but were rather due to the stability of the proteins.

## 3. Discussion

Subsets of intimin-lacking STEC strains express immunoglobulin-binding proteins that interact with human immunoglobulins [7]. These proteins are considered to be virulence factors; due to their abilities to bind the immunoglobulins IgA and/or IgG and/or the Fc fragment in a non-immune manner, they cause resistance of the producing bacteria to the human serum complement [3]. Such surface exposed proteins were identified in our previous study in intimin-negative STEC belonging to 14 serotypes and eight sequence types (STs), the most common being O91:H14/H^−^[H14], ST33, a frequent STEC serotype causing diarrhoea in adults in Germany [23]. The EibG protein is a putative afimbrial adhesin in STEC O91, as shown in our previous study [7]. This was demonstrated by the ability of cloned *eib*G to confer the typical chain-like adherence pattern to human and bovine intestinal epithelial cells [7]. Furthermore, the expression of the chain-like phenotype by wild-type bacteria from broth cultures supports the hypothesis that besides contributing to the host cell adherence, EibG may also be directly involved in chain formation by growing bacteria and, thus, in interbacterial contact [7].

In STEC, the EibG expression is upregulated by incubation under static conditions [8], as we also confirmed in this study. However, additional environmental conditions may also have an impact on EibG levels produced, as we demonstrate here by the extended spectrum of the experimental conditions. It is worth noting that despite the generally consistent effects of the particular conditions on the regulation of EibG expression, the EibG amount produced under the same conditions varied from strain to strain; this demonstrates a strain-specific EibG production.

In particular, we determined EibG expression by STEC under different growth conditions and protein stability. The proteins were detected on immunoblots following denaturing polyacrylamide gel electrophoresis; signal intensities were quantified. Using these conditions, EibG appeared as multimeric complexes with a dominant band of >250 kDa and also exhibited aggregates with lower molecular masses and lower signal intensities. EibG production with increased levels started at the late exponential and early stationary phases when incubated at 37 °C, while only faint signals were determined during the lag-phase and early exponential growth. This late induction of EibG synthesis in STEC is in agreement with studies of other proteins of the Eib family [2]. Notably, once expressed at high levels, the proteins are highly stable over a prolonged incubation time, this observation reported for the first time in our study.

Moreover, we investigated the influence of environmental regulatory effects on the upregulation of EibG synthesis in STEC strains and followed the composition of the growth media and culture conditions. The highest EibG expression was detected after growth in the complex media when cultivated statically. In the minimal media or yeast extract, the synthesis was considerably lower and the difference in the EibG production between shaking and non-shaking conditions was minimal, indicating a general downregulation under nutrient deprivation.

Furthermore, bacteria change in regards to metabolism and physiology when cell division is limited. Under stress conditions, they may produce additional and more virulent and regulatory proteins for survival such as quorum sensing regulators by impact of ethanolamine [24]. As an example, long polar fimbriae in *E. coli* O157:H7 are highly expressed in the late exponential phase at iron-limited and acid growth conditions [25]. Synthesis of EibG proteins in STEC is upregulated by several environmental factors. Temperature is one major factor, as increased temperatures triggered high EibG expression rates [8]. High levels of the protein were detected in the stationary phase during cultivation under static growth conditions in the complex media at 37 °C. Another environmental factor leading to EibG upregulation might be a reduced availability of oxygen. High protein levels were detected under microaerophilic and static growth conditions [8]. In contrast, pH played a minor role in regulation of EibG expression [8]. The above regulatory mechanisms might have implications for EibG expression in the human intestine during infection. The culture conditions that resulted in increased EibG synthesis, thus, indicate that the proteins may, indeed, be involved in virulence. Although the definitive biological functions of Eib proteins remain to be established, the multimerised proteins have been proposed to facilitate afimbrial adhesion potentially involved in the host-pathogen interactions and to serve as trimeric autotransporter adhesins in *E. coli* [22,26]. It is shown that immunoglobulin binding proteins have an important impact on the human complement system [2,27,28]. While the complement system plays an important role in protection against bacterial infection, strains display resistance to bactericidal activity of the complement system, contributing to their potential to survive, proliferate, resulting in infected hosts possibly becoming ill.

It is quite rare that virulence proteins are negatively regulated by shaking as we observed for EibG. Other adhesive proteins, such as fimbriae or pili, are upregulated primarily under shaking culture conditions [29,30]. Bacterial interactions with target cells, tissues, and other bacteria are mediated by such adhesive proteins [29,30]. The biological adhesions are induced by non-covalent, yet specific interactions between protein and receptor, and these processes might be affected by mechanical forces. The adhesion of type 1 fimbriae is strengthened under increasing mechanical stress conditions, and biological bonds are, therefore, tightened.

In conclusion, we show that several environmental factors have an impact on EibG expression in STEC, with static cultivation in the complex media resulting in a profound upregulation. High EibG expression levels were determined during the late stationary growth phase. Once expressed, EibG exhibits a high stability over extended incubation periods. The up- and downregulation of Eib protein synthesis in STEC seems to apply either to a variety of *eib* genes and their Eib products in non-pathogenic *E. coli* strains and this process is strain-specific.

## 4. Experimental Section

### 4.1. Strains and Culture Conditions

EibG-positive STEC strains (Table 1) were obtained from the strain collection of the Institute for Hygiene, Westfälische Wilhelms-University Münster and University Hospital of Münster, and from the Robert Koch Institute, Wernigerode, Germany. The *E. coli* strains ECOR2 and ECOR9 harbouring *eib*F and the genes *eib*A, *eib*C, *eib*D, and *eib*E, respectively, were obtained from the Reference Culture Collection [4]. Strains were screened for the presence of *eib* genes by PCR as described [8].

Single colonies grown on LB agar plates were pre-cultured in tubes with LB broth (Roth, Karlsruhe, Germany) under constant shaking conditions of 180 rpm at 37 °C. Based on overnight cultures, bacteria were inoculated at a 1:100 dilution followed by shaking or static incubation at 37 °C as indicated. Cultures were prepared in sterile flasks containing growth media such as LB broth, the yeast extract medium (HM; consisting of 0.2% (*w*/*v*) yeast extract and 2% (*w*/*v*) glucose in 20 mM phosphate buffer, pH 7.2), and the minimal medium (M9; 1.0% (*w*/*v*) glucose, 20 mM NH_4_Cl, 9 mM NaCl, 10 mM MgSO_4_, 1 mM CaCl_2_ in 20 mM phosphate buffer, pH 7.2). Media ingredients were purchased from Roth, Karlsruhe, Germany. When indicated, tetracycline (Roth, Karlsruhe, Germany) was added at a concentration of 20 µg/mL. After incubation, bacteria were harvested by centrifugation at 3000 rpm for 10 min at 4 °C. Pellets were resolved in sterile distilled water and stored at −20 °C until use.

### 4.2. Immunoblot Analysis and Signal Quantification

For lysis, cells were exposed to ultrasonic disintegration for 5–10 min. Protein amounts in each sample were determined photometrically using a modified Lowry protein determination method [31]. Finally, SDS-loading buffer was added and the protein suspensions were heated to 99 °C for 5–10 min prior to separation, using sodium dodecyl sulphate polyacrylamide (10%) gel electrophoresis (SDS-PAGE) under reducing conditions in a mini slab gel apparatus (MiniProtean3 cell system; Bio-Rad, Munich, Germany). Proteins were transferred onto polyvinylidene difluoride (PVDF) membranes (Immobilon-P; Roth, Karlsruhe, Germany) using a semi-dry blotting system (Roth, Karlsruhe, Germany) at 100 mA for 60 min. The membranes were blocked in Tris-buffered saline (TBS) containing 0.1% Tween 20 (TBST) and 1% (*w*/*v*) non-fat dry milk powder for 60 min. To detect EibG proteins, membranes were incubated with horseradish peroxidase (HRP)-conjugated human IgG Fc fragment at a concentration of 100 ng/mL (Jackson ImmunoResearch Laboratories, West Grove, PA, USA) with shaking for 16 h as described [8]. Following three TBST washes, signals were visualised using a chemiluminescence enhancement kit (SuperSignal West Pico; Thermo Scientific, Bonn, Germany) according to the manufacturer’s instructions for a chemiluminescence imager (Chemi Doc XRS, Bio-Rad, Munich, Germany) using a charge-coupled device (CCD) camera. The molecular protein masses were determined using the Precision Plus Protein Western C standard (Bio-Rad, Munich, Germany), and the signals of the standard proteins were visualised using HRP-labelled StrepTactin antibody (Bio-Rad, Munich, Germany) and chemiluminescence enhancement kit reaction. Eib protein signals were quantified using densitometry, and the linear range was determined semi-quantitatively in serial dilutions [32]. Determination of signal intensities was carried out by computerised integration of the signals using QUANTITY ONE and IMAGE LAB software (Bio-Rad, Munich, Germany). For analysis and comparison of data originating from different gel runs, the highest EibG signals were defined as 1.0 and the intensities of the dilutions were calculated proportionately as means. Variations in repeated SDS-PAGE runs were expressed as standard deviations of the means (±SD).

### 4.3. Phenotypic Characterization

The phenotype of the cultures was inspected in Erlenmeyer flasks after growth under shaking and static cultivation conditions in LB, yeast extract medium and minimal medium as indicated at 37 °C. Documentation occurred with a digital camera.

In order to analyse chain formation and aggregates, approximately 2 × 10^8^ cells of EibG-producing strains were fixed to the surface of glass slides (Langenbrinck, Emmendingen, Germany) by heating, and cells were stained with 0.1% crystal violet solution (Merck, Darmstadt, Germany) and inspected by light microscopy (Axio Imager.A1; Zeiss, Oberkochen, Germany). For microscopy, objectives of 40- and 100-fold magnification were used. Microscopic images were documented using a digital microscope camera with digital computer connections. 

### 4.4. Peptide Mass Fingerprinting

The corresponding protein bands separated by SDS-PAGE were subjected to in-gel tryptic digestion. Peptides obtained were analysed by matrix-assisted laser desorption/ionization mass spectrometry (MALDI-MS; QStar, AB Sciex, Concord, ON, Canada). Data resulting from peptide mass fingerprinting were matched by ProFound, MS-Fit, and Mascot.

## Figures and Tables

**Table 1:** STEC and reference strains used in this study.

Strain No.	Serotype ^a^	*eib* Genes	*eib* Subtype
4798/97-1	O146:H21	*eib*G	α
7140/96	O91:H^−^ [H14]	*eib*G	α
2875/96	O91:H14 [H14]	*eib*G	α
393/98	O91:H^−^ [H14]	*eib*G	α
1809/00	O91:H14 [H14]	*eib*G	α
01/E243	O91:H^−^ [H14]	*eib*G	α
6705/95	OR:H14 [H14]	*eib*G	α
99-02787	OR:H10	*eib*G	α
0520/99	Ont:H30	*eib*G	γ
3671/97	O91:H^−^ [H14]	*eib*C, *eib*G	α
172/98	OR:H^−^	*eib*C, *eib*G	α
6561/95	Ont:H^−^ [H14]	*eib*C, *eib*G	α
ECOR9	Ont:H^−^	*eib*A, *eib*C *eib*D, *eib*E	-
ECOR2	Ont:H32	*eib*F	-

^a^ [H14], strains harbour *fli*C H14.
